# Presentation of an Auditory Training Protocol Applied in Children with Central Auditory Processing Disorder

**DOI:** 10.1590/2317-1782/e20240022en

**Published:** 2025-02-10

**Authors:** Nádia Giulian de Carvalho, Mariana Venâncio Silveira Pereira, Maria Francisca Colella-Santos

**Affiliations:** 1 Programa de Pós-graduação em Saúde, Interdisciplinaridade e Reabilitação, Departamento de Desenvolvimento Humano e Reabilitação – DDHR, Faculdade de Ciências Médicas – FCM, Universidade Estadual de Campinas – UNICAMP - Campinas (SP), Brasil.; 2 Graduação em Fonoaudiologia, Universidade Estadual de Campinas – UNICAMP - Campinas (SP), Brasil.; 3 Departamento de Desenvolvimento Humano e Reabilitação e Centro de Investigação em Pediatria – DDHR, Faculdade de Ciências Médicas – FCM, Universidade Estadual de Campinas – UNICAMP - Campinas (SP), Brasil.

**Keywords:** Children, Hearing, Hearing Tests, Auditory Perception, Rehabilitation

## Abstract

**Purpose:**

To present an auditory training protocol in children with Central Auditory Processing Disorder (CAPD).

**Methods:**

The study included nine children aged from 9 to 12, with five females and four males. Only children with auditory thresholds within the normal range and bilateral type A tympanometric curves were selected. Initially, a behavioral assessment of Central Auditory Processing (CAP) was conducted, and a self-perception questionnaire was administered. Subsequently, eight sessions of auditory training were conducted following a defined protocol with four specific activities per session, aimed at training distinct auditory skills. In a third phase, a new CAP behavioral assessment was carried out, and the questionnaire was reapplied.

**Results:**

The quantitative analysis of the pre- and post-training behavioral tests showed statistically significant improvements in the Left Dichotic Digit Test (DDT), the Left Competing Dissyllable Test (SSW), the Left Synthetic Sentence Identification with Ipsilateral Competing Message Test (SSI), and the Random Gap Detection Test (RGDT). An improvement in auditory behavioral perception of the participants was also observed, as indicated by the self-perception questionnaire responses.

**Conclusion:**

Although the auditory training protocol did not result in complete normalization in the Central Auditory Processing (CAP) Behavioral Assessment tests, an improvement in the auditory skills of binaural integration, figure-ground and temporal resolution of participants was observed, as well as in their personal perception of these abilities.

## INTRODUCTION

Central Auditory Processing (CAP) is an essential process of the central nervous system (CNS) and refers to the efficiency and effectiveness with which the CNS uses auditory information, including a set of auditory skills required for the detection, analysis, association, and interpretation of sound information. Each behavioral test is designed to assess a specific auditory skill, thus evaluating different areas and functions of the Central Auditory Nervous System (CANS)^(1,2)^. Lesions or immaturity in the CANS pathways can lead to alterations in one or more auditory skills, resulting in difficulties in auditory information processing. This condition is called Central Auditory Processing Disorder (CAPD) and is referred to in the literature as a clinical entity, identified in ICD 10 as other abnormal auditory perceptions (H93.2)^(2)^.

CAPD represents a significant clinical challenge, affecting a considerable portion of the population, although with varying prevalence. In the United States, the estimated prevalence of CAPD is 2% to 5%; in the United Kingdom, 0.5% to 1% in the general population and 5.1% in children who have difficulty understanding speech in noisy environments; and in India, the estimated prevalence is 3.2%^(3-6)^. CAPD can impact several aspects of child development, including language, learning and social interaction^(7)^. With the diagnosis of CAPD, Auditory Training (AT) is the main direct intervention strategy in the clinical setting.

AT addresses a set of fundamental acoustic conditions, with tasks to activate the auditory system and related systems in order to change the neural basis and auditory behaviors^(8)^. Then, when considering the plasticity of the CNS, neuroplasticity can be induced through varied experiences and stimuli, which helps improve the synaptic efficiency, increase neural density, and induce cognitive and behavioral changes^(9)^.

The adherence to fundamental principles is essential for optimizing the changes induced by Auditory Training (AT). One of the most critical aspects is the subject’s attention, since the effectiveness of changes in the CNS depends directly on the patient’s level of attention during the presentation of stimuli. In addition, the tasks should stimulate the retention of information in memory, using techniques such as positive reinforcement, adequate frequency and repetition. Another crucial aspect is the gradual progression of the difficulty level of the tasks, ensuring that the patient is constantly challenged but not overwhelmed, in addition to the patient’s active participation in the process^(10)^. These principles aim to ensure that the activities are encouraging and effective, promoting significant advances in patient rehabilitation.

In this context, the creation of innovative tools and strategies has become a priority for professionals in the field. AT personalization to adjust it to patient needs and capabilities, is a crucial step to increase its effectiveness. It involves not only customizing auditory activities, but also integrating multidisciplinary strategies, which may include language therapy strategies, metalanguage, cognitive strategies, and home and classroom accommodations^(11)^. The goal is to create a holistic therapeutic environment that addresses all aspects of CAPD, resulting in a more comprehensive and lasting recovery.

The relevance of Computer-Based Auditory Training (CBAT) has been emphasized. It consists of acoustic tasks presented through computer interfaces, such as software, materials, and websites, especially designed to develop specific auditory skills. These can vary in terms of control of auditory stimuli. The possibility of controlling the sound intensity in decibels during acoustic tasks may allow more intense and precise training, adjusting the intensities to each ear individually. This is particularly feasible through Acoustically Controlled Auditory Training (ACAT)^(10)^.

Although there is still no consensus in the literature on the most effective form or protocol of Auditory Training (AT), studies have reported promising results with different training approaches^(12-15)^. The effectiveness of these interventions is commonly assessed through behavioral reassessments of auditory skills and electrophysiological examinations. Recently, the use of self-perception questionnaires has been recommended as indicators of auditory behaviors, which complement diagnostic tests^(16)^.

Despite the advances, the literature still lacks studies that integrate and compare the results of traditional behavioral assessments of CAP with data obtained through self-perception questionnaires. This gap is particularly critical because the lack of integration between these types of assessment can limit the understanding of the real impact of AT. Behavioral data can provide an objective view of the progress, while patient perceptions can offer insights into the underlying changes and their practical relevance.

Therefore, this study aims to fill this gap by proposing an auditory training protocol that was tested in children with CAPD. This study uses an intra-subject comparison approach to analyze both behavioral and self-perception data, providing a holistic and detailed assessment of the effects of AT.

## METHODS

This analytical interventional longitudinal study was approved by the institution’s Research Ethics Committee under report 2.041.609.

The inclusion criteria for the sample were: subjects aged 8 to 12 years; hearing thresholds within normal standards^(17)^; bilateral type A tympanometry curve; diagnosis of CAPD based on at least two altered tests^(18)^; and signed informed consent form (ICF) and assent form. Participants who did not attend all eight proposed acoustically controlled AT sessions and/or who did not complete the Behavioral Reassessment of CAP were excluded from this study.

The study sample had 10 subjects, who were selected from a speech therapy service at a teaching clinic; of these, 9 met the inclusion criteria. Thus, the final sample consisted of 9 subjects aged 9 to 12 years: 5 female and 4 male subjects.

The procedures were performed at a teaching clinic at a public university and were divided into three stages:

1. In the first stage, a behavioral assessment of Central Auditory Processing (CAP) was performed and a self-perception questionnaire (SPQ) was applied by a researcher;
2. In the second stage, eight auditory training sessions were conducted by another researcher;
3. In the third stage, a behavioral reassessment of CAP was performed and the self-perception questionnaire was reapplied by the researcher from the first stage.

The procedures that comprised the first and third stages were:

1. Speech by White Noise Test (SWNT): The normality criterion was ≥70% correct answers and a difference between the speech recognition index (SRI) and the speech/noise (S/N) ratio of less than 20%^(19)^.
2. Dichotic Digit Test (DDT) in the binaural integration stage: The normality criterion was ≥95% correct answers for the right ear (RE) and the left ear (LE)^(19)^.
3. Staggered Spondaic Word (SSW) test: The normality criterion was ≥90% correct answers for both ears^(19)^.
4. Synthetic Sentence Identification-Ipsilateral Competing Message (SSI-ICM): In the monotonic stage, ≥70% in the F/F ratio -10dB and ≥60% in the F/F ratio -15dB were used as reference values^(19)^.
5. Nonverbal Dichotic Test (NVDT): The normality criterion for Free Attention (FA) was defined as 10 to 14 correct answers, and for Right-Directed Listening (RDL) and Left-Directed Listening (LDL), more than 23 correct answers^(19)^.
6. Random Gap Detection Test (RGDT): The normality criterion was the average of the four sound frequencies ≤10 ms^(20)^.
7. Self-Perception Questionnaire (SPQ): This questionnaire is part of an online CAP screening battery named AudBility and contains 12 questions with scores ranging from 1 to 5 using a Likert scale (Chart 1). The researcher read the questions and selected the answer provided by the child. The response options are: always (1 point), frequently (2 points), sometimes (3 points), rarely (4 points), and never (5 points). The total score is the sum of the scores for all questions, ranging from 12 to 60 points, and the higher the score (between 45 and 60), the better the subject’s self-perception^(21)^.

**Chart 1:** Self-perception questionnaire (SPQ)

**CHILD VERSION**
**Always (5 points) / Frequently (4 points) / Sometimes (3 points) / Rarely (2 points) / Never (1 point)**
1. You are in a classroom or in an environment where people are talking.
**Do you have difficulty listening or understanding what the teacher is saying?**
2. The teacher or someone else is talking too fast to you.
**Do you have difficulty understanding what the teacher just said?**
3. The teacher or someone else is giving you spoken instructions (explanations).
**Do you have difficulty following spoken instructions?**
4. The teacher or someone else is talking to you in a quiet environment.
**Do you have difficulty listening and understanding the words clearly without changing any letter?**
5. When the teacher or a friend is talking to you.
**Do you feel that sometimes you hear well and sometimes you don’t?**
6. You are in the classroom or the schoolyard and someone calls your name.
**Do you have difficulty understanding where the sound is coming from?**
7. The teacher or someone else is talking to you.
**Do you ask this person to repeat what he or she said?**
8. You are in the classroom.
**Do you get distracted easily?**
9. Last year at school.
**Did you have learning difficulties?**
10. You are doing an activity.
**Do you have trouble focusing?**
11. When you are in the classroom or at home.
**Do people tell you that you are daydreaming or inattentive?**
12. When you are at school or at home.
**Are you disorganized??**

In the second stage of the study, the auditory training was conducted using a therapeutic intervention protocol. This protocol was based on activities available on the website www.afinandoocerebro.com.br, involving verbal and non-verbal tasks. However, to ensure accuracy and control the intensity of sound stimuli, the stimuli were presented in an acoustically controlled manner, using TDH39 headphones, a 2-channel audiometer (AC 40-Interacoustics) connected to a laptop from the institution to access the stimuli. This method is known as Acoustically Controlled Auditory Training (ACAT), and all sessions were conducted at the university’s teaching clinic, in a controlled environment equipped for this purpose. The sound intensity was adjusted to 50dB SL above the average of the tonal thresholds at the frequencies of 500Hz, 1KHz, and 2KHz.

A closed protocol with four activities per session was applied, with each activity to train a specific auditory skill, once a week, at the teaching clinic (Chart 2). The parameters of the games, such as signal/noise ratio and intensity, were not changed during the sessions. However, despite the closed protocol, a criterion of minimum correct answers of 70% in each activity was established for the participant to advance to the next stage of the training.

**Chart 2:** Auditory training protocol

**SKILLS**	**SESSION 1**	**SESSION 2**	**SESSION 3**	**SESSION 4**
**Figure-ground**	Restaurant	Treasure Hunt	Distorted Voice / Guess	Treasure Hunt
**(Monotonic listening)**	Level 1	Level 1	Audio	Level 2
**Temporal resolution**	Tunnel	Kids Horns	Time Perception	Drops
	Level 1	Level 1	Level 1	Level 1
**Binaural integration/ separation**	Binaural Separation of Numbers	Binaural Separation of Numbers	Binaural Integration of Digits	Young Wizards
	Level 1	Level 2	Level 1	Level 1
**Temporal Ordering/ Prosody**	Acting Class	Loud and Low Sound	Frequency Interval 5a	Acting Class
	Level 1	Level 2	Audio	Level 2
	**SESSION 5**	**SESSION 6**	**SESSION 7**	**SESSION 8**
**Figure-ground (Dichotic task)**	Restaurant	Treasure Hunt Level 3	Distorted Voice / Phrases Audio	Treasure Hunt
	Level 2			Level 4
**Temporal resolution**	Time Perception	Count the Sounds	Time Perception	Hard Horns
	Level 2	Audio	Level 3	Level 1
**Binaural integration**	Binaural Integration of Digits	Young Wizards	Binaural Integration of Digits	Young Wizards
	Level 2	Level 2	Level 3	Level 3
**Temporal Ordering/ Prosody**	Musical Score	Temporal Ordering	Hard Horns	Kids Horns
	Level 1	Audio	Level 2	Level 3

In the game named “Restaurant,” the subject assumes the role of a waiter in a restaurant. The task is to memorize the orders of customers, with several competitive stimuli that challenge concentration and memory. Then, on another screen, the player must read and/or say the order options correctly.

In the game named “Treasure Hunt,” the subject must pay attention to the main message and select the correct figure from four options that will appear in a chain. During the game, there will always be one or more stories competing with the sound of the main sentence, increasing the difficulty of listening.

In the audios “Distorted Voice,” the subject is challenged to identify the places mentioned by the speaker, who provides clues spoken in a distorted voice. In addition, in the sentence task, the player must repeat only the words that begin with a specific letter.

In the game named “Tunnel,” the challenge is to count how many moments of silence intervals are heard during the sound of rain. The player must pay attention to these moments of silence that occur intermittently amid the constant sound of rain.

In the games “Kids Horns” and “Hard Horns,” the challenge at level 1 is to count how many times the player heard the horn and then select the traffic light on the street that has the same number of cars. From level 2 onwards, the player must identify the correct order in which the horns are presented.

In the game named “Time Perception,” the subject must count how many double sounds he or she identified in each sequence of seven sounds.

In the game named “Drops,” the subject must, at certain times, simply count how many drops he or she heard. At other times, the player must identify the correct sequence, observing the number of drops in each set, and distinguish between low and high-pitched drops.

In the game named “Binaural Separation of Numbers,” the subject hears four numbers, two in each ear. At level 1, the player must repeat only the numbers heard in the right ear, while at level 2, the player must repeat only the numbers heard in the left ear.

In the game named “Binaural Integration of Digits,” the subject hears different numbers in each ear. At level 1, the player must repeat the numbers heard in both ears, and at subsequent levels, the numbers that he or she did not hear.

In the game named “Young Wizards,” the subject pays attention to the question regarding the preferences of little wizards that will appear in each round and answers using the four options presented in both ears.

In the game named “Acting Class,” the subject listens to the audio and must identify the emotion in the speaker’s voice and select a figure with a facial expression that corresponds to the emotion in the voice (happy, sad, angry, amazed, scared, insecure, and unmotivated).

In the game named “Loud and Low Sound,” the subject observes the strength of the sound that each character produces and must identify the order in which the sound produced by each character is presented (loud, medium, or low).

In the audio “Frequency Interval 5a,” the subject listens to two sounds and must say whether the sound went up or down.

In the audio “Count the Sounds,” the subject listens to a sequence of sounds with small interruptions. With each series, the intervals between them become smaller. The subject must say how many sounds he or she heard.

**Statistical analysis:** SPSS V20, Minitab 16, and Excel Office 2010 software tools were used in statistical analysis. A significance level of 0.05 (5%) was adopted. Results with statistical significance were highlighted in bold, and those that showed a trend towards significance were marked with an asterisk (*). The Shapiro-Wilks test (N<30) indicated the absence of normal distribution. Therefore, nonparametric tests were used. The chi-square test was used to analyze the relative frequency of classifications (normal/altered) of the CAP tests. The Wilcoxon test was used to compare the quantitative results before and after the intervention and analyze the questions and the total score of the questionnaire.

## RESULTS

In the analysis of the relative frequency distribution regarding the classification of the tests applied in the behavioral assessment before and after training of auditory skills (Table 1), a trend towards a statistically significant difference was observed in the Dichotic Digit Test (DDT) in the left ear.

**Table 1:** Distribution of relative frequency of the classification before and after auditory training tests

	Pre			Post		p-value
	N		%	N	%	
SSI F/F -10dB	Altered	3	33.3%	2	22.2%	0.353
	Normal	6	66.7%	7	77.8%	
SSI F/F -15dB	Altered	3	33.3%	2	22.2%	0.353
	Normal	6	66.7%	7	77.8%	
RGDT	Altered	3	42.9%	0	0.0%	0.077
	Normal	4	57.1%	8	100%	
SSW-RE	Altered	5	62.5%	4	50.0%	0.343
	Normal	3	37.5%	4	50.0%	
SSW-LE	Altered	8	100%	5	62.5%	0.100
	Normal	0	0.0%	3	37.5%	
DDT-RE	Altered	6	66.7%	4	44.4%	0.242
	Normal	3	33.3%	5	55.6%	
DDT-LE	Altered	8	88.9%	4	44.4%	0.061*
	Normal	1	11.1%	5	55.6%	
NVDT-FA	Altered	4	44.4%	2	22.2%	0.244
	Normal	5	55.6%	7	77.8%	
NVDT-RDL	Altered	4	44.4%	2	22.2%	0.244
	Normal	5	55.6%	7	77.8%	
NVDT-LDE	Altered	5	55.6%	3	33.3%	0.242
	Normal	4	44.4%	6	66.7%	
SWNT-RE	Altered	1	11.1%	1	11.1%	0.529
	Normal	8	88.9%	8	88.9%	
SWNT-LE	Altered	1	11.1%	1	11.1%	0.529
	Normal	8	88.9%	8	88.9%	

Chi-Square Test

* Tendency toward statistical significance

**Caption:** SSI: Synthetic Sentence Identification-Ipsilateral Competing Message; F/F: Main Message-Competitive Message Ratio; SWNT: Speech by White Noise Test; DDT: Dichotic Digit Test; SSW: Staggered Spondaic Word; NVDT: Nonverbal Dichotic Test; FA: free attention; RDL: right-directed listening; LDE: left-directed listening; RE: right ear; LE: left ear; RGDT: Random Gap Detection Test

The analysis of the quantitative results of the tests applied in the behavioral assessment before and after training of auditory skills (Table 2) showed that there were statistically significant results in the DDT in the left ear, in the SSW in the left ear, in the SSI in the F/F -10dB and F/F -15dB ratios in the left ear, and in the RGDT.

**Table 2:** Distribution of quantitative results before and after auditory training tests

			Mean	Median	Standard deviation	N	CI	p-value
SWNT	RE	Pre	86.2%	92.0%	10.4%	9	6.8%	0.188
		Post	90.7%	96.0%	12.0%	9	7.8%	
	LE	Pre	88.9%	92.0%	9.5%	9	6.2%	0.287
		Post	92.0%	96.0%	9.8%	9	6.4%	
DDT	RE	Pre	91.8%	92.5%	4.1%	9	2.7%	0.208
		Post	94.4%	95.0%	5.1%	9	3.3%	
	LE	Pre	83.1%	85.0%	6.9%	9	4.5%	**0.028**
		Post	92.4%	95.0%	6.0%	9	3.9%	
SSW	RE	Pre	79.7%	81.3%	14.3%	8	9.9%	0.249
		Post	85.3%	88.8%	11.8%	8	8.1%	
	LE	Pre	68.6%	75.0%	21.6%	8	15.0%	**0.042**
		Post	81.1%	87.5%	14.2%	8	9.8%	
NVDT – FA	RE	Pre	12.44	13.0	2.74	9	1.79	0.306
		Post	11.56	12.0	1.74	9	1.14	
	LE	Pre	11.00	10.0	3.16	9	2.07	0.400
		Post	11.89	12.0	1.96	9	1.28	
	Errors	Pre	0.556	0.0	0.726	9	0.475	0.739
		Post	0.556	0.0	1.014	9	0.662	
NVDT – RDL	RE	Pre	19.00	23.0	5.96	9	3.89	0.128
		Post	22.33	23.0	2.92	9	1.90	
	LE	Pre	4.44	1.0	6.02	9	3.94	0.225
		Post	1.44	1.0	2.55	9	1.67	
	Errors	Pre	0.556	0.0	0.726	9	0.475	0.257
		Post	0.222	0.0	0.441	9	0.288	
NVDT – LDE	RE	Pre	3.89	1.0	4.46	9	2.91	0.172
		Post	1.56	1.0	2.30	9	1.50	
	LE	Pre	19.67	22.0	5.02	9	3.28	0.125
		Post	22.00	23.0	3.16	9	2.07	
	Errors	Pre	0.444	0.0	0.726	9	0.475	1.000
		Post	0.444	0.0	1.014	9	0.662	
SSI F/F -10dB	RE	Pre	78.9%	90.0%	20.3%	9	13.2%	0.157
		Post	85.6%	90.0%	15.9%	9	10.4%	
	LE	Pre	75.6%	80.0%	22.4%	9	14.6%	**0.038**
		Post	83.3%	90.0%	22.9%	9	15.0%	
SSI F/F -15dB	RE	Pre	68.9%	70.0%	23.2%	9	15.1%	0.063*
		Post	80.0%	80.0%	17.3%	9	11.3%	
	LE	Pre	72.2%	70.0%	22.8%	9	14.9%	0.059*
		Post	78.9%	90.0%	21.5%	9	14.0%	
RGDT		Pre	10.54	10.0	5.51	7	4.08	**0.028**
		Post	4.61	4.3	2.33	7	1.73	

The Wilcoxon test

* Tendency toward statistical significance

**Caption:** SSI: Synthetic Sentence Identification-Ipsilateral Competing Message; F/F: Main Message-Competitive Message Ratio; SWNT: Speech by White Noise Test; DDT: Dichotic Digit Test; SSW: Staggered Spondaic Word; NVDT: Nonverbal Dichotic Test; FA: free attention; RDL: right-directed listening; LDE: left-directed listening; RE: right ear; LE: left ear; RGDT: Random Gap Detection Test; GAP (ms): Interstimulus interval in millisecond

Table 3 shows the comparison between pre-training (Q1) and post-training (Q2) of auditory skills for each question and the total score of the self-perception questionnaire. In the total score, an improvement was observed in the auditory behavior, according to the perception of the subjects.

**Table 3:** Distribution of the relative frequency of the self-perception questionnaire, considering the two questionnaire application times

		Mean	Median	Standard deviation	N	CI	p-value
Question 1	Q1	2.89	3,0	1.17	9	0.76	0.139
	Q2	3.78	4,0	0.83	9	0.54	
Question 2	Q1	2.22	2,0	1.30	9	0.85	0.319
	Q2	2.89	3,0	1.05	9	0.69	
Question 3	Q1	2.67	3.0	1.58	9	1.03	0.131
	Q2	3.67	4.0	1.50	9	0.98	
Question 4	Q1	4.33	5.0	1.41	9	0.92	0.854
	Q2	4.44	5.0	1.33	9	0.87	
Question 5	Q1	3.33	3.0	1.41	9	0.92	0.914
	Q2	3.22	3.0	1.39	9	0.91	
Question 6	Q1	2.33	2.0	1.50	9	0.98	0.105
	Q2	3.78	5.0	1.64	9	1.07	
Question 7	Q1	2.33	2.0	1.32	9	0.86	0.389
	Q2	3.00	3.0	1.32	9	0.86	
Question 8	Q1	2.22	2.0	1.30	9	0.85	0.238
	Q2	2.78	3.0	1.48	9	0.97	
Question 9	Q1	2.00	2.0	1.32	9	0.86	0.796
	Q2	2.33	2.0	1.58	9	1.03	
Question 10	Q1	2.11	2.0	1.36	9	0.89	0.066*
	Q2	3.33	3.0	1.32	9	0.86	
Question 11	Q1	2.56	2.0	1.74	9	1.14	0.395
	Q2	3.11	3.0	1.27	9	0.83	
Question 12	Q1	2.00	1.0	1.73	9	1.13	0.136
	Q2	3.22	3.0	0.97	9	0.63	
Total score	Q1	30.67	28.0	12.31	9	8.04	**0.042**
	Q2	40.22	41.0	7.53	9	4.92	

* Tendency toward statistical significance

Table 4 shows the characterization of the subjects and the classification of Central Auditory Processing Disorder (CAPD) before and after training of auditory skills. The type of alteration in Central Auditory Processing that was present in all cases was decoding.

**Table 4:** Characterization of subjects in terms of sex, age, and classification of Central Auditory Processing Disorder (CAPD) before and after auditory training

**Subjects**	**Sex**	**Age**	**CAPD classification**	
			**Pre-training**	**Post-training**
S1	Female	9 years	Decoding and Organization	Decoding and organization
S2	Male	10 years	Decoding and nonverbal gnosis impairment	Decoding
S3	Male	10 years	Decoding and coding	Decoding and coding
S4	Female	11 years	Decoding, organization, and nonverbal gnosis impairment	Decoding
S5	Female	12 years	Decoding and nonverbal gnosis impairment	Decoding and nonverbal gnosis impairment
S6	Male	11 years	Decoding, organization, and nonverbal gnosis impairment	Decoding
S7	Male	9 years	Decoding, coding, and nonverbal gnosis impairment	**Normalized**
S8	Female	10 years	Decoding, organization, and nonverbal gnosis impairment	Decoding
S9	Female	11 years	Decoding, coding, and nonverbal gnosis impairment	Decoding, organization, and nonverbal gnosis impairment

## DISCUSSION

The approach adopted in this study integrated the findings of the behavioral evaluation of Central Auditory Processing (CAP) with subjective measurements of the auditory behavior, obtained with the self-perception questionnaire of children diagnosed with Central Auditory Processing Disorder (CAPD). This methodology was selected to comprehensively evaluate the auditory training protocol.

The analysis of the relative frequency distribution (Table 1) found an increase in the “Normal” classification in the tests of the evaluation battery. Although it was not statistically significant, this increase was particularly significant in the Dichotic Digit Test, in which a statistical trend was observed on the left side after auditory training. Interestingly, the “Altered” and “Normal” classifications were equal in the right and left ears. This finding was confirmed in the analysis of the quantitative distribution (Table 2), in which the average percentage of correct responses increased in the left ear from 83.1% to 92.4%.

The considerable improvement in the auditory performance of the left ear observed in this study highlights the effective relationship between the trained skills, particularly binaural integration, and their corresponding improvements. The protocol applied in this study incorporated the activities named “Young Wizards” and “Binaural Integration of Digits,” designed to strengthen the binaural integration skill, which is essential for speech understanding in noisy environments. However, it is important to note that, with the current data, we cannot determine which of these activities made the most significant contribution to the results obtained. This study highlights the importance of training, although the individual contributions of each activity remain indistinct. Such improvement shows an optimization in the use of the neural connections already established, a phenomenon supported by the paradigm of dichotic stimuli presentation known as Dichotic Interaural Intensity Difference (DIID)^8^.

Regarding the analysis of the quantitative results of tests (Table 2), in addition to the previously discussed DDT, statistically significant differences were observed in the SSW, SSI, and RGDT tests. In the SSW test, despite the statistically significant improvement, the performance of subjects did not reach what was expected for this age group. This finding suggests that the auditory training protocol may need specific adjustments or a longer intervention period. It is important to emphasize that the SSW test requires binaural integration with a high linguistic load and involves sequencing and rapid changes in auditory attention. Persistent difficulties in the SSW test may also indicate the need for additional strategies focused on sequencing capacity and auditory attention to meet the linguistic and cognitive demands of this task. In addition, the association between performance in the SSW test and possible comorbidities with other neurodevelopmental disorders emphasizes the importance of referral to a multidisciplinary evaluation.

The findings from the SSW test are in line with those of a previous study^(22)^, which also found a statistically significant difference between the pre- and post-auditory training assessments in the SSW test (p-value <0.001), although the mean percentage of correct answers did not reach the normality criterion, that is, it confirms the idea that, even with the presence of statistical gains, reaching the normality criterion is a challenge that may not be overcome exclusively with AT.

In the RGDT test, a statistically significant improvement was obtained in the children’s ability to discriminate two acoustic stimuli in short intervals. Specifically, the minimum time required for this auditory discrimination decreased considerably from 10.54 ms to 4.61 ms (p=0.028), which indicates a significant advance in auditory temporal perception. This test is multifaceted, requiring an attentional focus to discern between the presentation of one or two sounds^(23)^. In addition, the ability to detect rapid transitions in sound stimuli is important for speech perception, as it facilitates the identification of subtle phonetic elements and can significantly influence speech understanding^(23,24)^. Therefore, based on this improvement, it is recommended to incorporate activities such as Tunnel, Kids Horns, Time Perception, and Drops in auditory training programs focused on temporal resolution ability. It was not possible to determine which specific activity is the most effective; however, the set of these activities resulted in significant improvements in temporal resolution ability.

The results of the SSI test, used to assess the figure-ground ability, revealed significant improvements in the monotonic stage. Notably, there was a statistical improvement in the F/F ratios -10 dB for the left ear (p=0.038) and a trend towards F/F -15dB (p=0.059 for the left ear). These findings show the neural plasticity of the CNS to form new synaptic connections and thus improve the auditory ability to understand speech in the presence of competitive noise^(23)^. Based on these results, it is suggested that specific auditory training activities with competitive stimuli, such as Restaurant and Treasure Hunt, should be incorporated. The implementation of these activities all together has proven to be particularly effective, suggesting that the synergy between different tasks can enhance changes in the auditory skills of participants.

It is important to emphasize that the main objective of auditory training is not to simply normalize the scores of the tests that make up the Central Auditory Processing assessment, but rather improve the access to auditory information and adapt auditory behavior in challenging contexts. Although we observed improvements in test scores and routinely compare pre- and post-auditory training performance in clinical practice, the focus should be on the subject’s functional ability to deal with everyday situations in which hearing is challenged. Auditory training interventions are performed to provide patients with better listening strategies, facilitating speech understanding in noisy environments and improving the quality of life of individuals with CAPD.

The results of the self-perception questionnaire (Table 3) indicate a significant improvement after the completion of the auditory training. The mean total score improved from 30.67 before training to 40.22 after training, reaching statistical significance with a p-value of 0.042. This increase in the score reflects a decrease in the frequency of hearing difficulties reported by the children, since a higher score in the questionnaire is directly proportional to a better self-perception of auditory performance.

It is important to highlight the relevance of the questionnaire used in this analysis, especially when considering each question individually. The most significant change, with a statistical trend, in the perception of participants occurred in question 10, which assesses the difficulty the subjects experienced in maintaining attention during an activity. A previous study using the same questionnaire adopted in this study, found a statistically significant difference in question 10 when comparing children with high and low academic performance^(16)^.

Comparatively, previous studies show that Brazilian children with typical development report self-perception of their auditory abilities with mean scores ranging from 44 to 46 points^(21,25)^. This comparison highlights the similarity of the post-training scores of the participants in this study to the performance of their peers with typical development, suggesting auditory training effectively improves the subjects’ perception of their own auditory abilities.

In our study, the significant increase in the mean total score in the second application of the questionnaire (Q2) showed a parallel improvement to the “Normal” classification found in the CAP behavioral assessment after auditory training. This positive evolution reinforces the results of a previous study^(26)^, which also reported consistent improvements in CAP behavioral tests and in self-perception questionnaires after the auditory training. Although the results did not reach the level of complete normality, the trend of improvement is evident both in the CAP behavioral parameters and in the participant self-perception after training. In addition, previous studies, in which the questionnaires were completed by the children’s caregivers, also indicated functional improvements in hearing after training, according to the responses of the guardians^(23,27)^. These collective response patterns suggest that auditory training can be an effective intervention not only from an objective perspective, but also from the subjective experience of children and parents or guardians. It highlights the importance of including subjective measures together with objective assessments to comprehensively evaluate the benefits of auditory training.

Prior to auditory training, the initial analysis of this study revealed that all participants (100%) had “decoding” difficulties as indicated in Table 4. Limitations associated with this specific category typically involve challenges in understanding messages in noisy environments, discriminating between similar sounds, decomposing the acoustic components of speech, and difficulties that may result in spelling errors^(28)^. Impacted auditory skills include auditory closure, figure-ground ability for verbal sounds, as well as binaural separation/integration and temporal resolution.

After the auditory training intervention, participants S2, S4, S6, S7, and S8 reached normative levels in some tests, resulting in a reduction in the number of altered categories of CAPD. However, “decoding” difficulties persisted in a large proportion of the sample (88.88%) in the second assessment. This pattern was also observed in other studies – one demonstrated that 90.5% of the subjects evaluated were diagnosed with “decoding” CAPD in the CAP Behavioral Assessment^(28)^ and another found a prevalence of 52.17% for this type of alteration^(26)^.

The persistence of “decoding” difficulties highlights the complexity of CAPD and the importance of multifaceted therapeutic approaches that address both specific auditory skills and compensatory strategies to improve auditory comprehension, especially in noisy environments. It also reflects the need for continuous assessment of patient progress and personalized adjustments to the protocol to achieve the best possible results.

The results obtained in this study highlight the contribution of the implemented auditory training protocol, with improvements in both auditory skills and auditory behavior of the participants. Also, the potential of standardized protocols should be noted, as it can be valuable tools in contexts that require collective interventions, such as public health services that treat a significant number of children awaiting rehabilitation for CAPD. Despite the usefulness of standardized protocols, the importance of individualized therapeutic planning whenever possible should be emphasized in order to address the specific needs of each patient and adjustments in the home and school contexts^(11)^.

A previous study that used the same digital platform Afinando o Cérebro, illustrates the advantage of adapting auditory training activities to the hearing deficiencies identified in each individual^(29)^. Although our study used a uniform protocol, the literature reinforces the value of personalized approaches, which can be more effective in addressing the particularities of every CAPD case^(29)^. In addition, it is important to highlight the role of subjective assessments performed by patients when evaluating the impact of auditory training. Although objective measures are crucial for assessing changes in auditory processing, the subjective perceptions of patients offer valuable insights into the benefits of training in their daily lives. Also, significant neural changes can often occur before behavioral improvements are observable. Therefore, subjective reports of improved auditory experiences may indicate neural improvements that have not yet fully manifested in behavioral tests. Such perceptions can guide early therapeutic adjustments and provide additional validation of treatment success, reinforcing the importance of considering both types of feedback – objective and subjective – for a comprehensive assessment of the effects of auditory training^(30)^.

Therefore, these findings highlight the need for continued investigation focused on the development of specific protocols for the different categories of CAPD. The goal is to provide targeted interventions to address the difficulties that are inherent to each type of alteration.

This study had limitations that should be considered. First, the small sample size. In addition, the follow-up period was relatively short, which may not allow an adequate assessment of the long-term effects of auditory training. Regarding the tests used in our study, although comprehensive, they may not cover all auditory processing mechanisms or detect subtle changes in specific skills, suggesting the need to include a wider range of tests.

Considering the limitations above, future studies should consider larger samples and add tests that assess a wider range of auditory skills, providing a better understanding of the interventions and effectiveness of personalized strategies, combining behavioral assessments and self-perception questionnaires applied before and after auditory training to assess the impact of such customization on the therapeutic process and treatment results.

## CONCLUSION

Although the auditory training protocol did not result in complete normalization in the Behavioral Assessment tests of Central Auditory Processing (CAP), the activities helped improve the auditory skills of binaural integration, figure-ground ability, and temporal resolution of the participants, as well as their personal perception of these skills.
