# Supplementary material for: Presentation of an Auditory Training Protocol Applied in Children with Central Auditory Processing Disorder
Source: Codas. 2025 Feb 10;37(2):e20240022. doi: 10.1590/2317-1782/e20240022en (PMC11813180; doi:10.1590/2317-1782/e20240022en)
Supplement: Translation

# Translation: Apresentação de um Protocolo de treinamento auditivo aplicado em crianças com Transtorno do Processamento Auditivo Central

**DOI:** 10.1590/2317-1782/e20240022pt

**Authors:** Nádia Giulian de Carvalho, Mariana Venâncio Silveira Pereira, Maria Francisca Colella-Santos

**Affiliations:** 1 Programa de Pós-graduação em Saúde, Interdisciplinaridade e Reabilitação, Departamento de Desenvolvimento Humano e Reabilitação – DDHR, Faculdade de Ciências Médicas – FCM, Universidade Estadual de Campinas – UNICAMP - Campinas (SP), Brasil.; 2 Graduação em Fonoaudiologia, Universidade Estadual de Campinas – UNICAMP - Campinas (SP), Brasil.; 3 Departamento de Desenvolvimento Humano e Reabilitação e Centro de Investigação em Pediatria – DDHR, Faculdade de Ciências Médicas – FCM, Universidade Estadual de Campinas – UNICAMP - Campinas (SP), Brasil.

**Keywords:** Crianças, Audição, Testes Auditivos, Percepção Auditiva, Reabilitação

## Abstract

**Objetivo:**

Apresentar um protocolo de treinamento auditivo em crianças com Transtorno do Processamento Auditivo Central (TPAC).

**Método:**

Participaram do estudo nove crianças entre 9 e 12 anos, sendo cinco do sexo feminino e quatro do sexo masculino. Foram selecionadas apenas crianças com limiares auditivos dentro da normalidade e curva timpanométrica tipo A bilateralmente. Inicialmente, foi realizada uma avaliação comportamental do Processamento Auditivo Central (PAC) e aplicado um questionário de autopercepção. Posteriormente, foram conduzidas oito sessões de treinamento auditivo, seguindo um protocolo definido com quatro atividades específicas por sessão, visando o treinamento de habilidades auditivas distintas. Em uma terceira fase, realizou-se nova avaliação comportamental do PAC e o questionário foi reaplicado.

**Resultados:**

A análise quantitativa dos testes comportamentais pré e pós-treinamento mostrou melhorias estatisticamente significativas no Teste Dicótico de Dígitos (TDD) à esquerda, no Teste Dicótico de Dissílabos Alternados (SSW) à esquerda, no Teste de Identificação de Sentenças Sintéticas com Mensagem Competitiva Ipsilateral (SSI) à esquerda e no Teste de Detecção de Intervalo Aleatório (Random Gap Detection Test - RGDT). Observou-se também melhora na percepção comportamental auditiva dos participantes, conforme indicado pelas respostas ao questionário de autopercepção.

**Conclusão:**

Apesar de o protocolo de treinamento auditivo não ter resultado na normalização completa nos testes de Avaliação Comportamental do Processamento Auditivo Central (PAC), observou-se uma contribuição na melhoria das habilidades auditivas de integração binaural, figura-fundo e resolução temporal dos participantes, bem como em sua percepção pessoal dessas capacidades.

## INTRODUÇÃO

O Processamento Auditivo Central (PAC) é uma função essencial do sistema nervoso central (SNC) e refere-se à eficiência e eficácia com que o SNC utiliza a informação auditiva, incluindo um conjunto de habilidades auditivas necessárias para a detecção, análise, associação e interpretação das informações sonoras. Cada teste comportamental é projetado para avaliar uma habilidade auditiva específica, avaliando assim diferentes áreas e funções do Sistema Nervoso Auditivo Central (SNAC)^(1,2)^. Lesões ou imaturidade nas vias do SNAC podem levar a alterações em uma ou mais habilidades auditivas, resultando em dificuldades no processamento da informação auditiva. No Brasil, essa condição é denominada Transtorno do Processamento Auditivo Central (TPAC) e é referido na literatura como uma entidade clínica, identificada no CID 10 como outras percepções auditivas anormais (H93.2)^(2)^.

O TPAC representa um desafio clínico significativo, afetando uma parcela notável da população, embora com prevalências variadas. Nos Estados Unidos, a prevalência de TPAC foi estimada entre 2% e 5%; no Reino Unido, entre 0,5% e 1% na população geral e 5,1% em crianças que apresentam dificuldades para entender a fala em ambientes ruidosos e na Índia, a prevalência foi estimada em 3,2%^(3-6)^. O TPAC pode impactar diversos aspectos do desenvolvimento infantil, incluindo linguagem, aprendizado e interação social^(7)^. Diante do diagnóstico de TPAC, o Treinamento Auditivo (TA) surge como a principal estratégia de intervenção direta no cenário clínico.

O TA engloba um conjunto de condições e tarefas acústicas fundamentais para ativar o sistema auditivo e sistemas correlatos. Esse processo visa modificar a base neural e os comportamentos auditivos^(8)^. Assim, levando em consideração a plasticidade do SNC, a neuroplasticidade pode ser induzida por meio de experiências e estímulos variados. Isso contribui para a melhoria da eficiência sináptica, aumento da densidade neural e indução de mudanças cognitivas e comportamentais^(9)^.

Para otimizar as mudanças induzidas pelo Treinamento Auditivo (TA), é essencial aderir a princípios fundamentais. Um dos mais críticos é a atenção do indivíduo, pois a eficácia da modificação no SNC depende diretamente do nível de atenção do paciente durante a apresentação dos estímulos. Além disso, as tarefas apresentadas devem estimular a retenção de informações na memória, empregando técnicas como o reforço positivo, a frequência e repetição adequadas. Outro aspecto crucial é a progressão gradual na dificuldade das tarefas, garantindo que o paciente esteja constantemente desafiado, mas não sobrecarregado, além de sua participação ativa no processo^(10)^. Esses princípios visam garantir que as atividades sejam motivadoras e eficazes, promovendo avanços significativos na reabilitação dos pacientes.

Neste contexto, a criação de ferramentas e estratégias inovadoras tem se tornado uma prioridade para os profissionais da área. A personalização do TA, ajustando-o às necessidades e capacidades individuais de cada paciente, é um passo crucial para aumentar sua eficácia. Isso envolve não apenas a customização das atividades auditivas, mas também a integração de estratégias multidisciplinares, que podem incluir estratégias terapêuticas de linguagem, metalinguagem, estratégias cognitivas, de acomodações em casa e na escola^(11)^. O objetivo é criar um ambiente terapêutico holístico que aborde todos os aspectos do TPAC, resultando em uma recuperação mais abrangente e duradoura.

A relevância do Treinamento Auditivo Baseado em Computador (TABC) tem sido enfatizada. O TABC consiste em tarefas acústicas apresentadas através de interfaces computacionais, como softwares, materiais e websites, especificamente programadas para desenvolver habilidades auditivas específicas. Estas podem variar quanto ao controle dos estímulos auditivos. A possibilidade de controlar a intensidade da apresentação sonora em decibels durante as tarefas acústicas pode permitir um treinamento mais intenso e preciso, ajustando as intensidades para cada orelha individualmente. Isso é particularmente viável através do Treinamento Auditivo Acusticamente Controlado (TAAC)^(10)^.

Embora ainda não haja um consenso na literatura sobre a forma ou o protocolo mais eficaz de Treinamento Auditivo (TA), estudos têm relatado resultados promissores em várias abordagens de treinamento^(12-15)^. A eficácia dessas intervenções é comumente avaliada por meio de reavaliações comportamentais das habilidades auditivas e exames eletrofisiológicos. Recentemente, tem-se recomendado o uso de questionários de autopercepção como indicadores de comportamentos auditivos, que complementam os testes diagnósticos^(16)^.

Apesar dos avanços, a literatura ainda carece de estudos que integrem e comparem os resultados das avaliações comportamentais tradicionais do PAC com os dados obtidos por meio de questionários de autopercepção. Esta lacuna é particularmente crítica porque a falta de integração entre essas modalidades de avaliação pode limitar a compreensão do impacto real do TA. Dados comportamentais podem fornecer uma visão objetiva do progresso, enquanto as percepções dos pacientes podem oferecer insights adicionais sobre as mudanças subjacentes e sua relevância prática.

Portanto, este estudo visa preencher essa lacuna, propondo um protocolo de treinamento auditivo que será testado em crianças com TPAC. A pesquisa emprega uma abordagem de comparação intrassujeito para analisar tanto os dados comportamentais quanto os de autopercepção, proporcionando uma avaliação holística e detalhada dos efeitos do TA.

## MÉTODO

Este estudo foi aprovado pelo Comitê de Ética em Pesquisa da Instituição sob o número 2.041.609. Apresenta um delineamento analítico, intervencional e longitudinal.

Os critérios de inclusão na amostra foram: faixa etária entre 8 e 12 anos; limiares auditivos dentro dos padrões de normalidade^(17)^; curva timpanométrica tipo A bilateral; diagnóstico de TPAC baseado em pelo menos dois testes alterados^(18)^; e assinatura do Termo de Consentimento Livre e Esclarecido (TCLE) e do Termo de Assentimento Livre e Esclarecido (TALE). Foram excluídos deste estudo os participantes que não compareceram às oito sessões propostas de TA acusticamente controlado e/ou que não realizaram a Reavaliação Comportamental do PAC.

Amostra: Dez sujeitos foram selecionados de um serviço de assistência fonoaudiológica de uma clínica-escola, dos quais nove atenderam aos critérios de inclusão. Assim, a amostra final foi composta por nove sujeitos na faixa etária de 9 a 12 anos, sendo cinco do sexo feminino e quatro do sexo masculino.

Os procedimentos foram realizados em uma clínica-escola de uma universidade pública. O trabalho dividiu-se em três etapas:

1. Na primeira etapa, realizou-se a avaliação comportamental do Processamento Auditivo Central (PAC) e a aplicação do questionário de autopercepção (QAPAC) realizado por um pesquisador;
2. Na segunda etapa, conduziram-se oito sessões de treinamento das habilidades auditivas realizadas por outro pesquisador;
3. Na terceira etapa, procedeu-se à reavaliação comportamental do PAC e à reaplicação do questionário de autopercepção pelo pesquisador da primeira etapa.

Os procedimentos que compuseram a primeira e terceira etapa foram:

1. Teste de Fala com Ruído Branco (TFR): Adotou-se como critério de normalidade ≥70% de acertos e uma diferença entre o Índice de Reconhecimento de Fala (IRF) e a relação Fala/Ruído (F/R) inferior a 20%^(19)^.
2. Teste Dicótico de Dígitos (TDD) na etapa da integração binaural: O critério de normalidade estabelecido foi de acertos ≥ 95% para a orelha direita (OD) e a orelha esquerda (OE)^(19)^.
3. Teste Dicótico de Dissílabos Alternados (SSW): Estabeleceu-se como critério de normalidade ≥ 90% de acertos para ambas as orelhas^(19)^.
4. Teste de Identificação de Sentenças Sintéticas com Mensagem Competitiva Ipsilateral (SSI): Na etapa monótica, utilizou-se como valor de referência ≥70% na relação F/F -10dB e ≥60% na relação F/F -15dB^(19)^.
5. Teste Dicótico Não-Verbal (TDNV): Definiu-se como critério de normalidade na Atenção Livre (AL) de 10 a 14 acertos e para a Escuta Direcionada à Direita (EDD) e Escuta Direcionada à Esquerda (EDE), mais de 23 acertos^(19)^.
6. *Random Gap Detection Test* (RGDT): O critério de normalidade utilizado foi a média das quatro frequências sonoras ≤ 10 ms^(20)^.
7. Questionário de autopercepção (QAPAC): Este questionário, faz parte de uma bateria online de triagem do PAC chamada AudBility, contém 12 questões com escores variando de 1 a 5, baseados na Escala *Likert* (Quadro 1). O examinador leu as perguntas e selecionou a resposta dada pela criança. As opções de resposta são: sempre (1 ponto), frequentemente (2 pontos), algumas vezes (3 pontos), raramente (4 pontos) e nunca (5 pontos). O Escore Total é a soma das pontuações de todas as questões, variando de 12 a 60 pontos, ou seja, quanto maior a pontuação (entre 45 e 60), melhor a autopercepção do sujeito^(21)^.

**Quadro 1:** Questionário de autopercepção (QAPAC)

**VERSÃO CRIANÇA**
**Sempre (05 pontos) / Frequentemente (4 pontos) / Algumas vezes (3 pontos) / Raramente (2 pontos) / Nunca (1 ponto)**
1. Você está em uma sala de aula ou em um ambiente em que tem pessoas conversando.
**Você tem dificuldade para escutar ou entender o que a professora está falando?**
2. A professora ou uma pessoa está falando muito rápido com você.
**Você tem dificuldade para entender o que a professora falou?**
3. A professora ou uma pessoa está dando instruções (explicações) faladas para você.
**Você tem dificuldade para seguir as instruções faladas?**
4. A professora ou uma pessoa está falando com você em um ambiente silencioso.
**Você tem dificuldade para escutar e entender claramente as palavras sem trocar nenhuma letra?**
5. Quando a professora ou um amigo está falando com você.
**Você tem a sensação de que às vezes você ouve bem e às vezes não?**
6. Você está em sala de aula ou no pátio da escola e alguém chama seu nome.
**Você tem dificuldade para perceber de onde vem o som?**
7. A professora ou uma pessoa está falando com você.
**Você pede para repetir o que foi falado?**
8. Você está na sala de aula.
**Você fica distraído com facilidade?**
9. No ano passado na escola.
**Você teve dificuldades para aprender?**
10. Você está fazendo uma atividade.
**Você tem dificuldade para ficar atento?**
11. Quando você está em sala de aula ou em casa.
**As pessoas falam que você é sonhador ou desatento?**
12.Quando você está na escola ou em casa.
**Você é desorganizado?**

Na segunda etapa do estudo, realizou-se o treinamento das habilidades auditivas através de um protocolo de intervenção terapêutica. Esse protocolo foi elaborado a partir de atividades já disponíveis no website “www.afinandoocerebro.com.br”, envolvendo tarefas verbais e não-verbais. Contudo, para garantir a precisão e controlar a intensidade dos estímulos sonoros, os estímulos foram apresentados de maneira acusticamente controlada, utilizando fones de ouvido (modelo TDH39), audiômetro de dois canais (AC 40-Interacoustics) acoplado a um notebook da instituição para acesso aos estímulos. Esse método é conhecido como Treinamento Auditivo Acusticamente Controlado (TAAC), e todas as sessões foram conduzidas na clínica-escola da universidade, em um ambiente controlado e equipado para tal. A intensidade sonora foi ajustada para 50dB NS acima da média dos limiares tonais nas frequências de 500Hz, 1KHz e 2KHz.

Adotou-se um protocolo fechado de quatro atividades por sessão, cada uma direcionada para treinar determinada habilidade auditiva, uma vez por semana apenas na clínica-escola (Quadro 2). Os parâmetros dos jogos tais como: relação sinal/ruído e intensidade não foram modificados durante as sessões, entretanto, apesar do protocolo ser fechado, foi estabelecido um critério de acertos mínimos de 70% em cada atividade para que o participante avançasse para a próxima etapa do treinamento.

**Quadro 2:** Protocolo de treinamento das habilidades auditivas

**HABILIDADES**	**1ª SESSÃO**	**2ª SESSÃO**	**3ª SESSÃO**	**4ª SESSÃO**
**Figura-Fundo**	Restaurante	Caçada ao Tesouro	Voz Distorcida/	Caçada ao Tesouro
**(Escuta Monótica)**	Nível 1	Nível 1	Adivinha – áudio	Nível 2
**Resolução Temporal**	Túnel	Buzinas Kids	Percepção de Tempo	Gotas
	Nível 1	Nível 1	Nível 1	Nível 1
**Integração/ Separação Binaural**	Separação	Separação	Integração	Jovens Bruxos
	Binaural Números	Binaural Números	Binaural Dígitos	Nível 1
	Nível 1	Nível 2	Nível 1	
**Ordenação Temporal / Prosódia**	Aula de Teatro	Forte e Fraco	Frequência Intervalo 5a	Aula de Teatro
	Nível 1	Nível 2	– áudio	Nível 2
	**5ª SESSÃO**	**6ª SESSÃO**	**7ª SESSÃO**	**8ª SESSÃO**
**Figura-Fundo (tarefa dicótica)**	Restaurante	Caçada ao Tesouro	Voz Distorcida / Frases – áudio	Caçada ao Tesouro
	Nível 2	Nível 3		Nível 4
**Resolução Temporal**	Percepção de Tempo	Conte os Sons –	Percepção de Tempo	Buzinas Hard
	Nível 2	áudio	Nível 3	Nível 1
**Integração Binaural**	Integração Binaural Dígitos	Jovens Bruxos	Integração Binaural Dígitos	Jovens Bruxos
	Nível 2	Nível 2	Nível 3	Nível 3
**Ordenação**	Pauta Musical	Ordenação	Buzinas Hard	Buzinas Kids
**Temporal / Prosódia**				
	Nível 1	Temporal – áudio	Nível 2	Nível 3

No jogo “Restaurante”, o sujeito assume o papel de garçom em um restaurante. A tarefa é memorizar os pedidos dos clientes, enfrentando diversos estímulos competitivos que desafiam a concentração e a memória. Em seguida, em outra tela, o jogador deve ler e/ou dizer corretamente as opções de pedidos.

No jogo "Caçada ao Tesouro," o sujeito deve estar atento à mensagem principal, que se refere à figura correta a ser selecionada entre quatro opções que aparecerão em uma corrente. Durante o jogo, sempre haverá uma ou mais histórias competindo com o som da frase principal, aumentando a dificuldade de escuta.

Nos áudios “Voz distorcida”, o sujeito é desafiado a identificar os lugares mencionados pelo falante, que fornece dicas faladas com uma voz distorcida. Além disso, na tarefa de frases, o jogador deve repetir apenas as palavras que começam com uma letra específica solicitada.

No jogo "Túnel," o desafio é contar quantos silêncios são ouvidos durante o som da chuva. O jogador precisa estar atento aos momentos de silêncio que ocorrem intermitentemente em meio ao som constante da chuva.

No jogo “Buzinas Kids” e “Buzinas Hard” o desafio no nível 1 é contar quantas vezes o jogador ouviu a buzina e, em seguida, selecionar o semáforo na rua que possui o mesmo número de carros, a partir do nível 2 deverá identificar a ordem correta de apresentação das buzinas.

No jogo “Percepção de tempo” o sujeito deve contar quantos sons duplos identificou em cada sequência de sete sons.

No jogo “Gotas”, o sujeito deverá, em determinados momentos, apenas contar quantas gotas escutou. Em outros momentos, ele deve identificar a sequência correta, observando a quantidade de gotas em cada conjunto, além de distinguir entre gotas graves e agudas.

No jogo “Separação Binaural Números”, o sujeito escuta quatro números, dois em cada ouvido. No nível 1, deve repetir apenas os números ouvidos na orelha direita, enquanto no nível 2, deve repetir apenas os números ouvidos na orelha esquerda.

No jogo “Integração Binaural Dígitos”, o sujeito escuta números diferentes em cada orelha. No nível 1, deve repetir os números que ouviu em ambas as orelhas, e nos níveis subsequentes, deve dizer os números que não ouviu.

No jogo “Jovens Bruxos”, o sujeito presta atenção à pergunta referente as preferências dos bruxinhos que aparecerá em cada rodada e responde às quatro opções de respostas apresentadas em ambas as orelhas.

No jogo “Aula de Teatro”, o sujeito escuta o áudio e deve identificar a emoção transmitida pela voz do falante e identificar as figuras com as expressões faciais correspondentes com a emoção transmitida pela entonação da voz (feliz, triste, bravo admirado/assustado, inseguro e desmotivado).

No jogo “Forte e Fraco”, o sujeito observa a força do som que cada personagem produz, e deve identificar a ordem de apresentação do som produzido por cada personagem (forte, médio ou fraco).

No áudio “Frequência Intervalo 5a”, o sujeito escuta dois sons e deve dizer se o som subiu ou desceu.

No áudio “Conte os Sons”, o sujeito escuta uma sequência de sons com pequenas interrupções. A cada série os intervalos entre eles ficam menores. O sujeito deve dizer quantos sons ouviu.

**Análise Estatística:** Para a análise estatística, utilizaram-se os softwares SPSS V20, Minitab 16 e Excel Office 2010. Adotou-se um nível de significância de 0,05 (5%). Os resultados com significância estatística foram destacados em negrito, e aqueles que apresentaram tendência à significância, com um asterisco (*). O teste de Shapiro-Wilks (N<30) indicou a ausência de distribuição normal. Portanto, recorreu-se a testes não paramétricos. O teste de Qui-quadrado foi empregado para analisar a frequência relativa das classificações (normal/alterado) dos testes do PAC. O teste de Wilcoxon foi utilizado para comparar os resultados quantitativos pré e pós-intervenção, bem como para analisar as questões e o escore total do questionário.

## RESULTADOS

Na análise da distribuição de frequência relativa referente à classificação dos testes aplicados na avaliação comportamental pré e pós-treinamento das habilidades auditivas (Tabela 1), verificou-se tendência a diferença estatisticamente significativa no Teste Dicótico de Dígitos (TDD) na orelha esquerda.

**Tabela 1:** Distribuição da frequência relativa da classificação dos testes pré e pós-treinamento das habilidades auditivas.

	**Pré**		**Pós**			**p-valor**
	N		%	N	%	
SSI F/F -10dB	Alterado	3	33,3%	2	22,2%	0,353
	Normal	6	66,7%	7	77,8%	
SSI F/F -15dB	Alterado	3	33,3%	2	22,2%	0,353
	Normal	6	66,7%	7	77,8%	
RGDT	Alterado	3	42,9%	0	0,0%	0,077
	Normal	4	57,1%	8	100%	
SSW-OD	Alterado	5	62,5%	4	50,0%	0,343
	Normal	3	37,5%	4	50,0%	
SSW-OE	Alterado	8	100%	5	62,5%	0,100
	Normal	0	0,0%	3	37,5%	
TDD-OD	Alterado	6	66,7%	4	44,4%	0,242
	Normal	3	33,3%	5	55,6%	
TDD-OE	Alterado	8	88,9%	4	44,4%	0,061*
	Normal	1	11,1%	5	55,6%	
TDNV-AL	Alterado	4	44,4%	2	22,2%	0,244
	Normal	5	55,6%	7	77,8%	
TDNV-EDD	Alterado	4	44,4%	2	22,2%	0,244
	Normal	5	55,6%	7	77,8%	
TDNV-EDE	Alterado	5	55,6%	3	33,3%	0,242
	Normal	4	44,4%	6	66,7%	
TFR-OD	Alterado	1	11,1%	1	11,1%	0,529
	Normal	8	88,9%	8	88,9%	
TFR-OE	Alterado	1	11,1%	1	11,1%	0,529
	Normal	8	88,9%	8	88,9%	

Teste Qui-Quadrado

* Tendência à significância estatística

**Legenda:** SSI: Teste de Reconhecimento de Sentenças Sintéticas com Mensagem Competitiva Ipsilateral. F/F: Relação Mensagem principal-Mensagem competitiva, TFR: Teste de Fala com Ruído Branco; TDD: Teste Dicótico de Dígitos, SSW: Teste Dicótico de Dissílabos Alternados, TDNV: Teste Dicótico Não-Verbal; AL: Atenção Livre; EDD: Escuta direcionada à direita; EDE: Escuta direcionada à esquerda, OD: Orelha direita; OE: Orelha esquerda, RGDT: *Random Gap Detectioon Test*

Na análise dos resultados quantitativos dos testes aplicados na avaliação comportamental pré e pós-treinamento das habilidades auditivas (Tabela 2), verificou-se que houve resultados estatisticamente significantes no TDD à esquerda, no SSW à esquerda, no SSI nas relações F/F -10dB e F/F -15dB à esquerda, e no RGDT.

**Tabela 2:** Distribuição dos resultados quantitativos dos testes pré e pós-treinamento das habilidades auditivas

			**Média**	**Mediana**	**Desvio Padrão**	**N**	**IC**	**P-valor**
TFR	OD	Pré	86,2%	92,0%	10,4%	9	6,8%	0,188
		Pós	90,7%	96,0%	12,0%	9	7,8%	
	OE	Pré	88,9%	92,0%	9,5%	9	6,2%	0,287
		Pós	92,0%	96,0%	9,8%	9	6,4%	
TDD	OD	Pré	91,8%	92,5%	4,1%	9	2,7%	0,208
		Pós	94,4%	95,0%	5,1%	9	3,3%	
	OE	Pré	83,1%	85,0%	6,9%	9	4,5%	**0,028**
		Pós	92,4%	95,0%	6,0%	9	3,9%	
SSW	OD	Pré	79,7%	81,3%	14,3%	8	9,9%	0,249
		Pós	85,3%	88,8%	11,8%	8	8,1%	
	OE	Pré	68,6%	75,0%	21,6%	8	15,0%	**0,042**
		Pós	81,1%	87,5%	14,2%	8	9,8%	
TDNV – AL	OD	Pré	12,44	13,0	2,74	9	1,79	0,306
		Pós	11,56	12,0	1,74	9	1,14	
	OE	Pré	11,00	10,0	3,16	9	2,07	0,400
		Pós	11,89	12,0	1,96	9	1,28	
	Erros	Pré	0,556	0,0	0,726	9	0,475	0,739
		Pós	0,556	0,0	1,014	9	0,662	
TDNV – EDD	OD	Pré	19,00	23,0	5,96	9	3,89	0,128
		Pós	22,33	23,0	2,92	9	1,90	
	OE	Pré	4,44	1,0	6,02	9	3,94	0,225
		Pós	1,44	1,0	2,55	9	1,67	
	Erros	Pré	0,556	0,0	0,726	9	0,475	0,257
		Pós	0,222	0,0	0,441	9	0,288	
TDNV – EDE	OD	Pré	3,89	1,0	4,46	9	2,91	0,172
		Pós	1,56	1,0	2,30	9	1,50	
	OE	Pré	19,67	22,0	5,02	9	3,28	0,125
		Pós	22,00	23,0	3,16	9	2,07	
	Erros	Pré	0,444	0,0	0,726	9	0,475	1,000
		Pós	0,444	0,0	1,014	9	0,662	
SSI F/F -10dB	OD	Pré	78,9%	90,0%	20,3%	9	13,2%	0,157
		Pós	85,6%	90,0%	15,9%	9	10,4%	
	OE	Pré	75,6%	80,0%	22,4%	9	14,6%	**0,038**
		Pós	83,3%	90,0%	22,9%	9	15,0%	
SSI F/F -15dB	OD	Pré	68,9%	70,0%	23,2%	9	15,1%	0,063*
		Pós	80,0%	80,0%	17,3%	9	11,3%	
	OE	Pré	72,2%	70,0%	22,8%	9	14,9%	0,059*
		Pós	78,9%	90,0%	21,5%	9	14,0%	
RGDT		Pré	10,54	10,0	5,51	7	4,08	**0,028**
		Pós	4,61	4,3	2,33	7	1,73	

Teste de Wilcoxon

* Tendência à significância estatística

**Legenda:** SSI: Teste de Reconhecimento de Frases com Mensagem Competitiva. F/F: Relação Mensagem principal-Mensagem competitiva, TFR: Teste de Fala com Ruído Branco; TDD: Teste Dicótico de Dígitos, SSW: Teste Dicótico de Dissílabos Alternados, TDNV: Teste Dicótico Não-Verbal; AL: Atenção Livre; EDD: Escuta direcionada à direita; EDE: Escuta direcionada à esquerda, OD: Orelha direita; OE: Orelha esquerda, RGDT: *Random Gap Detection Test*; GAP (ms): Intervalo Interestímulos em milissegundos

Na Tabela 3, encontra-se a comparação entre os momentos pré (Q1) e pós (Q2) do treinamento das habilidades auditivas para cada questão e o escore total do questionário de autopercepção. Verificou-se no escore total uma melhora no comportamento auditivo na percepção dos sujeitos.

**Tabela 3:** Distribuição da frequência relativa do questionário de autopercepção, considerando os dois momentos de aplicação

		**Média**	**Mediana**	**Desvio Padrão**	**N**	**IC**	**P-valor**
Questão 1	Q1	2,89	3,0	1,17	9	0,76	0,139
	Q2	3,78	4,0	0,83	9	0,54	
Questão 2	Q1	2,22	2,0	1,30	9	0,85	0,319
	Q2	2,89	3,0	1,05	9	0,69	
Questão 3	Q1	2,67	3,0	1,58	9	1,03	0,131
	Q2	3,67	4,0	1,50	9	0,98	
Questão 4	Q1	4,33	5,0	1,41	9	0,92	0,854
	Q2	4,44	5,0	1,33	9	0,87	
Questão 5	Q1	3,33	3,0	1,41	9	0,92	0,914
	Q2	3,22	3,0	1,39	9	0,91	
Questão 6	Q1	2,33	2,0	1,50	9	0,98	0,105
	Q2	3,78	5,0	1,64	9	1,07	
Questão 7	Q1	2,33	2,0	1,32	9	0,86	0,389
	Q2	3,00	3,0	1,32	9	0,86	
Questão 8	Q1	2,22	2,0	1,30	9	0,85	0,238
	Q2	2,78	3,0	1,48	9	0,97	
Questão 9	Q1	2,00	2,0	1,32	9	0,86	0,796
	Q2	2,33	2,0	1,58	9	1,03	
Questão 10	Q1	2,11	2,0	1,36	9	0,89	0,066*
	Q2	3,33	3,0	1,32	9	0,86	
Questão 11	Q1	2,56	2,0	1,74	9	1,14	0,395
	Q2	3,11	3,0	1,27	9	0,83	
Questão 12	Q1	2,00	1,0	1,73	9	1,13	0,136
	Q2	3,22	3,0	0,97	9	0,63	
Escore total	Q1	30,67	28,0	12,31	9	8,04	**0,042**
	Q2	40,22	41,0	7,53	9	4,92	

* Tendência à significância estatística

Na Tabela 4, é possível verificar a caracterização dos sujeitos e a classificação do Transtorno do Processamento Auditivo Central (TPAC) nos momentos pré e pós-treinamento das habilidades auditivas. O tipo de alteração do Processamento Auditivo Central que esteve presente em todos os casos foi a de decodificação.

**Tabela 4:** Caracterização dos sujeitos quanto ao sexo, idade e classificação do Transtorno do Processamento Auditivo pré e pós treinamento das habilidades auditivas

**Sujeitos**	**Sexo**	**Idade**	**Classificação do TPAC**	
			**Pré- treinamento**	**Pós-treinamento**
S1	Feminino	9 anos	Decodificação e Organização	Decodificação e Organização
S2	Masculino	10 anos	Decodificação e Prejuízo GnósicoNão-Verbal	Decodificação
S3	Masculino	10 anos	Decodificação e Codificação	Decodificação e Codificação
S4	Feminino	11 anos	Decodificação, Organização e Prejuízo Gnósico Não-Verbal	Decodificação
S5	Feminino	12 anos	Decodificação e Prejuízo GnósicoNão-Verbal	Decodificação e Prejuízo GnósicoNão-Verbal
S6	Masculino	11 anos	Decodificação, Organização e Prejuízo Gnósico Não-Verbal	Decodificação
S7	Masculino	9 anos	Decodificação, Codificação e Prejuízo Gnósico Não-Verbal	**Normalizou**
S8	Feminino	10 anos	Decodificação, Organização e Prejuízo Gnósico Não-Verbal	Decodificação
S9	Feminino	11 anos	Decodificação, Codificação ePrejuízo Gnósico Não-Verbal	Decodificação, Organização,Prejuízo Gnósico Não-Verbal

## DISCUSSÃO

Neste estudo, adotou-se uma abordagem que integra os achados da avaliação comportamental do Processamento Auditivo Central (PAC) com medidas subjetivas do comportamento auditivo, sendo este último derivado do questionário de autopercepção das crianças diagnosticadas com Transtorno do Processamento Auditivo Central (TPAC). Essa metodologia foi escolhida para avaliar de forma abrangente o protocolo de treinamento auditivo.

Na análise da distribuição de frequência relativa (Tabela 1), constatou-se um aumento na classificação 'Normal' nos testes da bateria de avaliação. Embora não tenha sido estatisticamente significativo, esse aumento foi particularmente notável no Teste Dicótico de Dígitos, onde se observou uma tendência estatística do lado esquerdo após o treinamento auditivo. De forma interessante, as classificações 'Alterado' e 'Normal' se equipararam nas orelhas direita e esquerda. Este achado foi corroborado pela análise da distribuição quantitativa (Tabela 2), onde na orelha esquerda a média da porcentagem de acertos aumentou de 83,1% para 92,4%.

A melhora notável no desempenho auditivo da orelha esquerda, observada neste estudo, destaca a relação efetiva entre as habilidades treinadas, particularmente a integração binaural, e suas melhorias correspondentes. O protocolo aplicado incorporou as atividades denominadas Jovens Bruxos e Integração Binaural Dígitos, projetadas para fortalecer a habilidade de integração binaural, essencial para a compreensão da fala em ambientes ruidosos. No entanto, é importante notar que, com os dados atuais, não podemos determinar qual dessas atividades contribuiu mais significativamente para os resultados observados. Este estudo ressalta a importância do treinamento, embora as contribuições individuais de cada atividade permaneçam indistintas. Tal melhora demonstra uma otimização no uso das conexões neurais já estabelecidas, um fenômeno apoiado pelo paradigma de apresentação dos estímulos dicóticos conhecido como *Dichotic Interaural Intensity Difference* (DIID)^8^.

Em relação à análise dos resultados quantitativos dos testes (Tabela 2), observou-se, além do TDD, discutido anteriormente, diferenças estatisticamente significantes nos testes SSW, SSI e RGDT. No teste SSW, apesar da melhora estatisticamente significativa, o desempenho dos sujeitos não atingiu o esperado para esta faixa etária. Esse achado sugere que o protocolo de treinamento auditivo pode precisar de ajustes específicos ou de um período de intervenção mais prolongado. É importante ressaltar que o teste SSW exige integração binaural com alta carga linguística e envolve sequenciamento e mudanças rápidas na atenção auditiva. A persistência de dificuldades no teste SSW também pode indicar a necessidade de estratégias adicionais focadas na capacidade de sequenciamento e atenção auditiva para atender às demandas linguísticas e cognitivas dessa tarefa. Além disso, a associação entre o desempenho no teste SSW e possíveis comorbidades com outros transtornos do neurodesenvolvimento enfatiza a importância de encaminhamento para uma avaliação multidisciplinar.

Os achados no teste SSW estão alinhados com estudo prévio^(22)^, que também encontrou uma diferença estatisticamente significativa entre as avaliações pré e pós-treinamento auditivo no teste SSW (p-valor < 0,001), embora a média da porcentagem de acertos não tenha alcançado o critério de normalidade, ou seja, reforça a ideia de que, mesmo com a presença de ganhos estatísticos, alcançar o critério de normalidade é um desafio que pode não ser superado exclusivamente pelo TA.

No teste RGDT, obteve-se uma melhoria estatisticamente significativa na capacidade das crianças em discriminar dois estímulos acústicos em intervalos breves. Especificamente, o tempo mínimo necessário para essa discriminação auditiva diminuiu consideravelmente, passando de 10,54 ms para 4,61 ms (p=0,028), o que indica um avanço notável na percepção temporal auditiva. A importância desse teste é multifacetada, exigindo um foco atencional para discernir entre a apresentação de um ou dois sons^(23)^. Além disso, a habilidade de detectar transições rápidas em estímulos sonoros é importante para a percepção de fala, pois facilita a identificação de elementos fonéticos sutis e pode influenciar significativamente a compreensão do discurso^(23,24)^. Portanto, com base nesta melhoria recomenda-se a incorporação de atividades como Túnel, Buzinas Kids, Percepção de Tempo e Gotas em programas de treinamento auditivo com foco na habilidade de resolução temporal. Não foi possível determinar qual atividade específica é a mais eficaz; no entanto, observou-se que o conjunto dessas atividades resultou em melhorias significativas na habilidade de resolução temporal.

Os resultados do teste SSI, utilizado para avaliar a habilidade de figura-fundo, revelaram melhorias significativas na etapa monótica. Notadamente, houve uma melhoria estatística nas relações F/F -10dB para a orelha esquerda (p=0,038) e uma tendência para F/F -15dB (p=0,059 à esquerda). Estes achados evidenciam a capacidade de plasticidade neural do SNC para formar novas conexões sinápticas e, assim, aprimorar a habilidade auditiva de compreender a fala em presença de ruído competitivo^(23)^. Com base nesses resultados, sugere-se a incorporação de atividades específicas de treinamento auditivo com estímulos competitivos, tais como Restaurante e Caçada ao Tesouro. A implementação dessas atividades em conjunto demonstrou ser particularmente eficaz, sugerindo que a sinergia entre diferentes tarefas pode potencializar mudanças nas habilidades auditivas dos participantes.

É importante destacar que o principal objetivo do treinamento auditivo não é simplesmente normalizar os escores dos testes que compõem a avaliação do Processamento Auditivo Central, mas sim melhorar o acesso à informação auditiva e adequar o comportamento auditivo em contextos desafiadores. Embora tenhamos observado melhorias nos escores dos testes e rotineiramente utilizamos tais comparações na prática clínica para comparar o desempenho pré e pós treinamento auditivo, o foco deve estar na capacidade funcional do indivíduo para lidar com situações do cotidiano em que a audição é desafiada. As intervenções do treinamento auditivo estão direcionadas para proporcionar aos pacientes melhores estratégias de escuta, facilitando a compreensão da fala em ambientes ruidosos e melhorando a qualidade de vida dos indivíduos com TPAC.

Os resultados do questionário de autopercepção, conforme demonstrado na Tabela 3, indicam um avanço significativo após a conclusão do treinamento auditivo. A média do escore total melhorou de 30,67 antes do treinamento para 40,22 após o treinamento, alcançando significância estatística com um p-valor de 0,042. Este aumento no escore reflete uma diminuição na frequência das dificuldades auditivas relatadas pelas crianças, uma vez que um escore mais alto no questionário é diretamente proporcional a uma melhor autopercepção do desempenho auditivo.

É fundamental destacar a relevância do questionário utilizado nesta análise, especialmente ao considerar cada questão individualmente. A mudança mais notável, com tendência estatística, na percepção dos participantes ocorreu na questão 10, que avalia a dificuldade em manter a atenção durante uma atividade. Um estudo anterior, que empregou o mesmo questionário adotado neste estudo, revelou uma diferença estatisticamente significativa na questão 10 ao comparar crianças com alta e baixa performance acadêmica^(16)^.

Comparativamente, estudos anteriores mostram que crianças brasileiras com desenvolvimento típico relatam uma autopercepção de suas habilidades auditivas com escores médios situando-se entre 44 e 46 pontos^(21,25)^. Essa comparação destaca a proximidade dos escores pós-treinamento dos participantes deste estudo com o desempenho de seus pares com desenvolvimento típico, sugerindo uma eficácia substancial do treinamento auditivo na melhoria da percepção das próprias habilidades auditivas dos sujeitos.

No estudo em questão, o aumento significativo da média do escore total na segunda aplicação do questionário (Q2) reflete uma melhoria paralela à classificação 'Normal' encontrada na avaliação comportamental do PAC após o treinamento auditivo. Essa evolução positiva reforça os resultados em estudo prévio^(26)^, que também relataram avanços consistentes em testes comportamentais do PAC e em questionários de autopercepção com o término do treinamento auditivo. Embora os resultados não tenham atingido o nível de normalidade completa, a tendência de melhoria é evidente tanto nos parâmetros comportamentais do PAC quanto na autopercepção dos participantes após o treinamento. Além disso, estudos anteriores, nos quais os questionários foram preenchidos pelos cuidadores das crianças, também indicaram melhorias funcionais na audição pós-treinamento, conforme as respostas dos responsáveis^(23,27)^. Estes padrões de resposta coletiva sugerem que o treinamento auditivo pode ser uma intervenção efetiva não apenas na perspectiva objetiva, mas também na experiência subjetiva das crianças e pais ou responsáveis. Isso ressalta a importância de incluir medidas subjetivas em conjunto com avaliações objetivas para avaliar de maneira abrangente os benefícios do treinamento auditivo.

Antes do treinamento auditivo, a análise inicial deste estudo revelou que todas as crianças participantes (100%) apresentaram dificuldades de 'decodificação' conforme indicado na Tabela 4. As limitações associadas a essa categoria específica geralmente envolvem desafios em compreender mensagens em locais barulhentos, discriminar entre sons similares, decompor os componentes acústicos da fala e dificuldades que podem resultar em erros de grafia^(28)^. As habilidades auditivas impactadas incluem fechamento auditivo, figura-fundo para sons verbais, bem como separação, integração binaural e resolução temporal.

Após a intervenção do treinamento auditivo, observou-se que os participantes S2, S4, S6, S7 e S8 alcançaram níveis normativos em alguns testes, resultando em uma redução no número de categorias alteradas do TPAC. Contudo, a dificuldade de 'decodificação' persistiu em uma grande parte da amostra (88,88%) na segunda avaliação. Este padrão é corroborado pelos achados de outros estudos, onde um demonstrou que 90,5% dos sujeitos avaliados foram diagnosticados com TPAC de 'decodificação' na Avaliação Comportamental do PAC^(28)^ e outro encontrou uma prevalência de 52,17% para este tipo de alteração^(26)^.

A persistência da dificuldade de 'decodificação' ressalta a complexidade do TPAC e a importância de abordagens terapêuticas multifacetadas que enderecem tanto as habilidades auditivas específicas quanto as estratégias compensatórias para melhorar a compreensão auditiva, especialmente em ambientes ruidosos. Isso também reflete a necessidade de uma avaliação contínua do progresso do paciente e ajustes personalizados no protocolo para alcançar os melhores resultados possíveis.

Os resultados obtidos neste estudo apontam para a contribuição do protocolo de treinamento auditivo implementado, evidenciando melhorias tanto nas habilidades auditivas quanto no comportamento auditivo dos participantes. É importante destacar o potencial dos protocolos padronizados, os quais podem ser de grande valia em contextos que demandam intervenções coletivas, como é o caso dos serviços públicos de saúde que lidam com um volume significativo de crianças aguardando reabilitação para o TPAC. Apesar da utilidade dos protocolos padronizados, deve-se enfatizar a importância de um planejamento terapêutico individualizado sempre que possível, a fim de abordar as necessidades específicas de cada paciente e ajustes no contexto doméstico e escolar^(11)^.

Pesquisa anterior, que utilizou a mesma plataforma digital "Afinando o Cérebro", ilustra bem a vantagem de adaptar as atividades de treinamento auditivo às deficiências auditivas identificadas em cada indivíduo^(29)^. Embora nosso estudo tenha seguido um protocolo uniforme, a literatura reforça o valor de abordagens personalizadas, que podem ser mais eficazes para atender às particularidades de cada caso de TPAC^(29)^. Além disso, é fundamental destacar o papel das avaliações subjetivas realizadas pelos pacientes ao avaliar o impacto do treinamento auditivo. Embora as medidas objetivas sejam cruciais para avaliar as mudanças no processamento auditivo, as percepções subjetivas dos pacientes oferecem insights valiosos sobre os benefícios do treinamento em suas vidas diárias. Importante ressaltar que, frequentemente, mudanças neurais significativas podem ocorrer antes que as melhorias comportamentais sejam observáveis. Por isso, relatos subjetivos de experiências auditivas melhoradas podem ser indicativos de avanços neurais que ainda não se manifestaram completamente em testes comportamentais. Tais percepções podem guiar ajustes terapêuticos precoces e fornecer uma validação adicional do sucesso do tratamento, reforçando a importância de considerar ambos os tipos de feedback – objetivo e subjetivo – para uma avaliação compreensiva dos efeitos do treinamento auditivo^(30)^.

Portanto, estes achados sublinham a necessidade de continuidade das pesquisas focadas no desenvolvimento de protocolos que sejam específicos para as diferentes categorias de TPAC. O objetivo é proporcionar uma intervenção mais direcionada às dificuldades inerentes a cada tipo de alteração.

Este estudo enfrentou limitações que devem ser consideradas. Primeiramente, o tamanho reduzido da amostra. Além disso, a duração do acompanhamento foi relativamente curta, o que pode não permitir a avaliação adequada dos efeitos de longo prazo do treinamento auditivo. Quanto aos testes utilizados, apesar de abrangentes, eles podem não abranger todos os mecanismos do processamento auditivo ou detectar mudanças sutis em habilidades específicas, sugerindo a necessidade de inclusão de uma gama mais ampla de testes.

Diante das limitações mencionadas, sugere-se para pesquisas futuras ampliar a amostra e adicionar testes que avaliem uma gama mais ampla de habilidades auditivas, proporcionando uma compreensão mais completa das intervenções e eficácia de estratégias personalizadas, combinando avaliações comportamentais e questionários de autopercepção, aplicados antes e depois do treinamento auditivo, para avaliar o impacto dessa customização no processo terapêutico e nos resultados do tratamento.

## CONCLUSÃO

Apesar de o protocolo de treinamento auditivo não ter resultado na normalização completa nos testes de Avaliação Comportamental do Processamento Auditivo Central (PAC), observou-se uma contribuição das atividades na melhoria das habilidades auditivas de integração binaural, figura-fundo e resolução temporal dos participantes, bem como em sua percepção pessoal dessas capacidades.
